# Urinary tract infections in healthy women: a revolution in management?

**DOI:** 10.1186/1471-2296-11-42

**Published:** 2010-05-26

**Authors:** Chris Del Mar

**Affiliations:** 1 School of Medicine, Faculty of Health Sciences, Bond University, Queensland 4229, Australia

## Abstract

**Background:**

Urinary infection in otherwise healthy women has largely been a straightforward matter of diagnosis by identifying bacteria in the urine, and then cure by appropriate antibiotics. Recent research has shown this to be over-simplified. Evaluation of methods of self-management of symptoms has been neglected.

**Discussion:**

Firstly trial data show that women with what used to called 'urethral syndrome' (urinary symptoms but sterile urine) obtain relief from antibiotics. Other trial data have shown a surprisingly large placebo effect from the resolution of symptoms among women who feel their care has been 'positive'. In addition, data published this month in *BMC Medicine *show that non-steroidal anti-inflammatory (NSAID) drugs provide symptom relief to women with conventional infections (positive urine bacteriology) as much as antibiotics.

**Conclusions:**

These recent findings provide an opportunity to consider how clinicians might change practice, and sets a new research agenda. We need to know (1) whether the effect of NSAIDs is replicable; (2) why some women in previous trials have had more symptoms if not treated with antibiotics sooner; (3) whether NSAIDs and antibiotics have an additive effect on relieving symptoms; (4) how we can harness the placebo effect better to assist out patients with this distressing and common complaint.

See research article http://www.biomedcentral.com/1741-7015/8/30

## Introduction

Urinary infections are very common among otherwise healthy women: a very high number experience symptoms and will have sought treatment before the age of 40. Among the aetiological actors, being sexually active seems to be the most important, both before and after menopause [1-3]. Diabetes is a well-known risk factor [4]. There is little that can be done to prevent urinary infections in healthy women. Therefore most attention is focussed on treating infections as they arise.

Although asymptomatic bacteriuria is a predictor for an increased risk of subsequently experiencing symptomatic urinary infections later, there is not regarded as a strong association enough to warrant screening [4] (except in antenatal care, recurrent infections or anatomical abnormality - not under consideration here).

### Diagnosis

Diagnosis has been considered straightforward in the past. The presence of bacteria on urine culture has been regarded as the gold standard. There has been discussion about the use of urine dipsticks (particularly with strips for testing for nitrites and leukocyte esterase) to guide treatment before results of cultures are returned. Urine dipsticks are useful for ruling out disease [5,6], and there has been a good argument for treating otherwise healthy women with antibiotics empirically if dipsticks are positive [5].

If there were no bacteria found on culture, an infective cause for the symptoms seemed unlikely. This led to the generation of a new term for 'culture-negative symptoms of urinary frequency, urgency and dysuria', called 'urethral syndrome', regarded as difficult to treat [7].

### Conventional treatment

Conventional treatment has principally focussed on eradicating bacterial causes. This seems on first consideration to be entirely logical: the urine is normally sterile, and symptoms of infection (frequency, and dysuria especially) are relieved after the bacteria have been cleared. Most organisms (and *Escherichia coli *among the faecal bacteria by far the most common) are responsive to cheap and available antibiotics.

Consequently, the focus of interest in the medical literature has been on selecting the appropriate antibiotic, the tension existing between ensuring the likelihood of effectiveness of the antibiotic, and minimising antibiotic resistance in the community.

This approach has been turned on its head by the surprising finding of a randomised controlled trial (RCT) in the recent past of women with symptoms of urinary infection, but negative findings for bacteria in the urine [8]. The RCT found benefit for symptoms with antibiotic over no antibiotics - even when conventional wisdom had held that they were not infected by bacteria (but had what might have been called 'urethral syndrome' in the past) [8].

On the other side of the coin, women with urinary infections randomised to delay taking antibiotics by two days suffer only about a day or two more of symptoms [9]. In other words, we can think of UTI as a spontaneously remitting disease, if we use symptoms (rather than sterility of the urine[10]) as the end-point, which is accelerated by the use of antibiotics.

A prognosis study has shown that the psychological state of the woman is important - those with 'somatisation' (exaggerated feelings of symptoms ) are likely to overcome their symptoms slower, while those who perceived the doctor to be positive about quickly overcoming symptoms recovered faster [9].

Like so much in medicine, the arena is messy, with many factors influencing recovery.

One thing is clear: whether treatment should be aimed at eliminating bacteria from the urine or not, certainly treatment of the symptoms warrants attention.

## The evidence

This is then a fertile time to think about using symptoms control rather than 'bacterial eradication' as an approach.

### Urinary alkalinisers self management

It has been traditional to use urinary alkalinisers in the past as symptomatic relief (by the simple expedient of taking sodium or potassium bicarbonate or other alkaline by mouth) [11]. This is remarkably poorly evaluated in the medical literature [12]. An observational study found symptom severity within each woman's illness was not associated with increases in urinary pH, suggesting alkalinising the urine is unlikely to reduce symptoms [12]. A factorial randomised trial which randomised yielded no difference to those randomised to advice to use bicarbonate or not [5].

### Cranberry juice self management

Although cranberry juice is a well used 'alternative and complementary medicine' for urinary tract infection, and there is weak and contradictory evidence it might act as a *preventive *agent [13], there is insufficient good quality trial evidence to support its use as a *treatment *[14]. One good RCT has been published recently to suggest that it is not [5].

### Non-steroidal anti-inflammatory (NSAIDs) drugs

These are commonly used for pain and inflammatory conditions, and, although NSAIDs have adverse effects, they are regarded as sufficiently safe to be available in most jurisdictions over-the-counter without prescription.

The pragmatic trial published this month in *BMC Medicine *by Bleidorn and colleagues [15] randomised antimicrobial treatment against symptomatic treatment (in the form of a non-steroidal anti-inflammatory drug) for urinary symptoms. The treatments were equivalent not just in the outcome of symptoms (which might have been predicted), but in the outcome of subsequent urinary infections. Why is this revolutionary? Because it turns on its head the notion that we should regard urinary infections as primarily an infectious process that needs sterilisation of the urine to effect resolution.

It used to be thought that urinary infections were sufficiently unusual and bizarre to requite extermination of the bacterial intruder. However two things have altered this approach. Firstly we recognise that some people (especially the elderly) often have bacteriuria with no ill effect, including no symptoms: treating them with antibiotics achieves only bacterial resistance. More recently we have come to realise that women who have urinary symptoms but no evidence of bacterial infection detected on urinary dip-stick - women who would have accordingly been denied antibiotics using traditional treatments - benefit symptomatically from antibiotics [8]. Guidelines have adjusted to this in some quarters[16] (but not all[17]).

## Pre-publication history

The pre-publication history for this paper can be accessed here:

http://www.biomedcentral.com/1471-2296/11/42/prepub

## Conclusions

### What does this study mean for clinicians?

The evaluation of a well-known drug with this new indication represents a new tool for the clinician. Here is something apparently *equally effective *as antibiotics for managing the symptoms of urinary infections in otherwise healthy women. It may be ued by clinicians as an adjunct to using antibiotics, or as a substitute for them (pending research confirmation - see below).

It will be interesting to see if word gets out to women in the community that their distressing symptoms can be alleviated by a simple purchase from the pharmacy or (in many countries) supermarket.

The study invites a reappraisal of the approach to many infections. Focussing on managing symptoms - once decried as 'symptom swatting' - rather than providing a 'cure' [18,19], may be a legitimate approach after all. Similar revolutions have taken place in other groups of disease, especially acute respiratory illnesses [20].

### What does this study mean for researchers?

Questions remain. Firstly many will want such a counter-intuitive finding to be replicated. Secondly we need to resolve the previous trial data showing that delaying antibiotics in many women with urinary symptoms resulted in longer endurance of symptoms. Third it would be useful to see if the two approaches - antibiotic 'cure' and NSAIDs 'symptom management' - are additive. Since they work by different mechanisms we might think that they might be, but it is possible that their effects eclipse each other, and any benefit of both over either is only marginal. This could be answered by an RCT with a factorial design (such as has been used in the area already [5]). Finally, the intriguing placebo effect that we are just beginning to understand described above [5], needs dissecting out in such a way that clinicians can harness it therapeutically.

## Authors' information

CDM is professor of primary care research at Bond University, Australia. He is also the coordinating editor of the Cochrane Collaboration's acute respiratory infection group.
